# Genesis of the Dawadi potassium nitrate deposit in Lop Nor, China

**DOI:** 10.1038/s41598-021-01278-3

**Published:** 2021-11-11

**Authors:** Yu Zhang, Lichun Ma, Kai Wang

**Affiliations:** 1 MNR Key Laboratory of Saline Lake Resources and Environments, Institute of Mineral Resources, Chinese Academy of Geological Sciences, Beijing, 100037 China; 2 China University of Geosciences (Beijing), Beijing, 100083 China

**Keywords:** Economic geology, Hydrology, Solid Earth sciences, Geochemistry, Geology

## Abstract

Nitrate deposits are rare worldwide, especially potassium nitrate deposits; furthermore, their genesis remains disputed. There is a rare salt-lake type potassium nitrate deposit in the Dawadi area of Lop Nor at the eastern margin of the Tarim Basin, and the ore bodies show coexisting solid and liquid phases. Additionally, there are large sulphate-type potash deposits in the adjoining Luobei Depression, south of the Dawadi area. To determine why there are two different types of potash deposits in adjacent depressions with similar climates, field geological surveys were conducted and samples collected. It was found that the Tertiary clastic layer at the periphery of the Dawadi deposit was rich in high-salinity brine, with nitrate contents of up to 495–16,719 mg/L, much higher than those in the Luobei Depression, 1–35 mg/L. Additionally, a type of deep hydrothermal (Ca–Cl) brine was found in the fault zones, with nitrate contents of up to 8044 mg/L, dozens of times greater than that of ordinary groundwater. Using comprehensive analysis and research, we concluded that the Dawadi and Luobei depressions belong to different hydrological systems with no connection between them; thus, the two deposits belong to different metallogenic systems. Furthermore, groundwater played an important role in the mineralization of the potassium nitrate deposit, and a deep source may have been an important source of the ore-forming materials. The fault system widely developed in Lop Nor provides favorable channels for deep hydrothermal recharge, and the groundwater and deep hydrothermal brine could provide the source for the nitrate mineralization in the Dawadi Depression through water–rock reactions.

## Introduction

**Figure 1:**
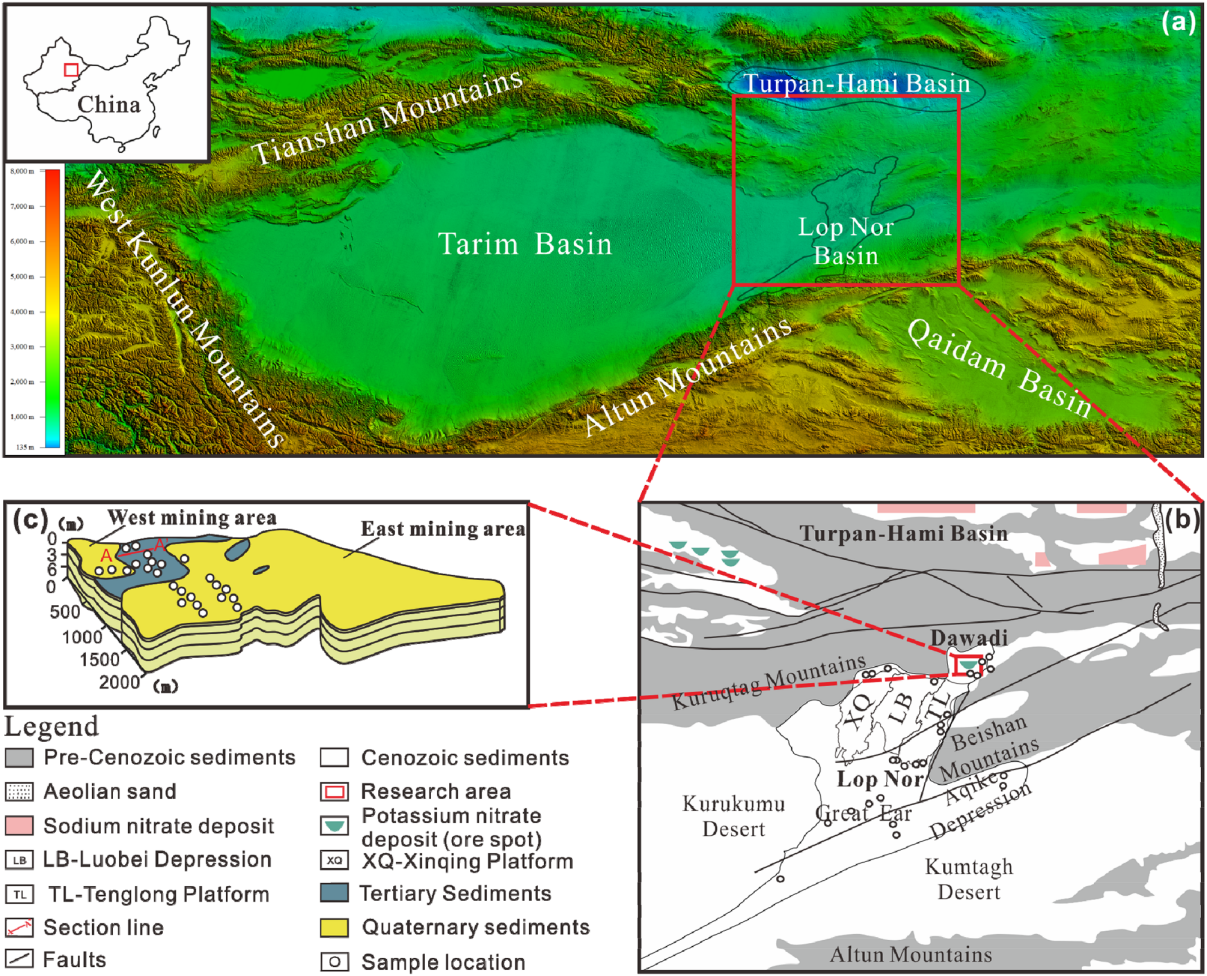
Tarim basin and its surrounding orogens (**a**); geological map and sampling points of the Turpan–Hami Basin-Lop Nor region (**b**)^4,20^; mining area of Dawadi and sampling points (**c**)^16^.

Because of the high solubility of nitrate, nitrate deposits are very rare globally, distributed only in extremely arid areas, such as the Atacama Desert in Chile, the Mojave Desert in the United States, the McMurdo Dry Valleys in Antarctica, and the Gobi Desert in the Turpan-Hami Basin of China^1^. Among them, the largest nitrate metallogenic belt, found in the Atacama region of Chile, is about 700 km long and 30 km wide at maximum, with reserves of 250 million tons^2,3^. In addition, from the Turpan-Hami Basin to the Lop Nor Basin, there is a sodium-nitrate metallogenic province in the Turpan-Hami area, and a potassium-rich nitrate metallogenic belt on the southern edge of the eastern Tianshan Mountains (Fig. 1), with cumulative reserves also of up to 250 million tons^4^. The origin of such giant nitrate deposits, especially the source of the nitrate, has been widely explored^2,5–8^.

Previous analytical determinations of nitrogen and oxygen isotopes in the world's major nitrate deposits have indicated that the δ^15^N[NO_3_^−^] values of nitrate deposits are generally close to atmospheric values^5–7^, and that the δ^18^O[NO_3_^−^] values are basically in the range of atmospheric δ^18^O_V-SMOW_, atmospheric deposition δ^18^O_V-SMOW_[NO_3_^−^], and δ^18^O_V-SMOW_[NO_3_^−^] of rainwater in the outer suburbs of Colorado^5,9–14^. Furthermore, the high Δ^17^O[NO_3_^−^] values in each deposit show significant oxygen isotope non-mass fractionation, suggesting that the NO_3_^−^ originated from atmospheric photochemical reactions^6,7,9,10^. In addition, the ^129^I value of the Atacama nitrate deposit is very similar to that of the deep subsurface sediment pore water, as well as that of the shales surrounding the mine, and δ^53/52^Cr also has distinct isotopic fractionation; therefore, Pérez-Fodich et al.^8^ suggested that water–rock interaction and leaching of groundwater with rocks around the basin provided an important material source of the deposit.

Lop Nor is one of the most important potassium-forming basins in China; however, the Luobei Depression, one of its sub-depressions, contains giant sulphate-type potassium-rich brine deposits and solid polyhalite [K_2_Ca_2_Mg(SO_4_)_4_·2H_2_O] deposits, whereas its adjacent area, Dawadi, contains a typical nitrate-type potash deposit (Fig. 1); the solid potash mineral is nitre (KNO_3_). These two basins are adjacent to each other and have similar climates. However, they have different types of potash deposits, which has attracted widespread attention from salt-lake geologists. Because of these recent advances^15,16^, available data are not sufficiently diagnostic of the hydrological relationship between these two depressions, let alone the relationship between these two different potash deposits. Based on the previous work, this study was conducted to systematically reorganize and investigate the characteristics of potassium nitrate deposits in Dawadi, and a large number of subsurface brine samples were collected at the periphery of the mine area. In addition, systematic investigation and sampling of salt springs and underground brines were carried out in Lop Nor and the Dawadi area, and they were compared with studies of other typical nitrate deposits. Thus, we could evaluate the relationships of the different potash deposits in the Dawadi and Luobei depressions and the genesis of the Dawadi potassium nitrate deposits.

## Regional geological background

The Lop Nor Basin is located at the north-eastern margin of the Tarim Basin, with the Kuruqtag Mountains to the north and the Altun Mountains to the south, and is under the control of Ruoqiang left-slip fractures and Kongqi River right-slip fractures, with north–south rigid rhombus-shaped spreading^17^ (Fig. 1). The formation and evolution of the Lop Nor Basin are closely related to the Himalayan Movement, which caused a violent north–south extrusion in the Tarim Basin Late from the Middle Pleistocene to Late Pleistocene^18^. As a result, the northern part of Lop Nor gradually uplifted, Lop Nor gradually dried up and disintegrated^19^, and a series of sub-depressions, such as the Luobei and Dawadi depressions, formed (Fig. 1). The coordinates of the Luobei Depression are 90°45'–91°20'E, 40°30'–41°10'N, and the potassium-rich brine deposits cover an area of about 1,300 km^2^^19^. The geographic coordinates of the potassium nitrate mining area in Dawadi are 91°44′E, 41°11′N; it is a small intramontane basin with an area of about 12 km^2^^1^. It belongs to the Paleozoic rift system of the Beishan Mountains and is adjacent to the Kawabulak and Xingdu-Kuruqtag faults^4^. The Oligocene–Miocene Peach-tree-garden Formation and Quaternary (Holocene) strata are exposed, and the Oligocene–Miocene strata are widely distributed, mainly as the basement of lacustrine chemical deposits, whereas the Holocene strata are mainly flood deposits, residual slope deposits, and lacustrine chemical deposits^15^, as shown in Fig. 1.

## Methods

In this study, salt spring, subsurface phreatic water, and solid samples were collected from the Dawadi Depression, Luobei Depression, the “Great Ear” lake area, and the perimeter of the Lop Nor Basin; the locations of the sampling points are shown in Fig. 1. GPS was used to locate sampling sites and record elevation data. Each solid sample was collected in two pieces, ensuring that the seal was intact to prevent the salt samples from absorbing moisture; liquid samples were collected in two bottles and sealed with tape immediately after collection to prevent evaporation under the extreme arid climate or leakage during transportation.

The samples were tested and analyzed at the National Geological Experiment and Test Center of the Chinese Academy of Geological Sciences (Beijing). Na^+^, K^+^, Mg^2+^, Ca^2+^, and SO_4_^2−^ contents of the samples were detected by plasma spectrometry (PE8300) based on the inspection method of GB 8538-2016 (PRC National Standard); the analytical errors were less than 0.2% for Ca^2+^, SO_4_^2−^ and Mg^2+^, and less than 0.5% for Na^+^ and K^+^. B, Li, Br, and I were detected by plasma mass spectrometry (PE300Q), based on the inspection method of GB 8538–2016 (PRC National Standard), with detection errors of less than 2%. Cl^−^, HCO_3_^−^, and NO_3_^−^ were detected by ion chromatography, based on the inspection method of DZG 20.01-1991 (PRC National Standard); the detection error was less than 0.2%.

## Results and discussion

### Geology of the deposits

The potassium nitrate deposit in Dawadi, as a salt-lake type deposit, shows solid–liquid phase coexistence. The deposit extends from northeast to southwest and is divided by a muddy crossbeam into two mining areas. Compared with the east mining area, the west mining area is better enclosed and has better deposit quality, with a concentrated phreatic brine ore body and solid-phase ore. In contrast, the solid-phase nitre (KNO_3_) in the east mining area only reaches the boundary grade, which is insufficient for exploitation. The brines in the liquid-phase potassium nitrate deposits of Dawadi mainly occurred in the intercrystalline pores of halite (NaCl) and salt cake (Na_2_SO_4_·10H_2_O). The chemical components were dominated by Cl^−^, Na^+^, NO_3_^−^, SO_4_^2−^, and K^+^ (Table 1). According to the water chemistry classification of Valyashko^21^, the brine in the mining area is of the sodium sulphate subtype. The west mining area contains a phreatic brine ore body; the water table is at about 0.94–1.20 m depth, the aquifer thickness is 2.20–4.67 m, and the mean salinity is 362.77 g/L. The east mining area contains a confined brine ore body with a water table depth of 1.12 m, aquifer thickness of 2.18 m, and salinity of 341.37 g/L^1^.

**Table 1:** Chemical compositions of brines of the potassium nitrate deposit in Dawadi.

		Ca^2+^ (g/L)	Mg^2+^ (g/L)	Na^+^ (g/L)	K^+^ (g/L)	HCO_3_^−^ (g/L)	NO_3_^−^ (g/L)	SO_4_^2−^ (g/L)	Cl^−^ (g/L)	B (mg/L)	Li (mg/L)	Br (mg/L)	I (mg/L)
The west mining area	Max	0.41	2.03	134.54	8.50	0.09	36.21	23.64	181.41	20.56	2.82	1.80	0.44
	Min	0.37	1.27	127.04	4.00	0.06	13.57	21.00	177.40	9.90	0.76	0.36	0.22
	Average	0.39	1.62	129.05	6.60	0.08	23.15	22.53	179.35	14.78	1.26	1.17	0.36
	Coefcient of variation	0.05	0.16	0.02	0.23	0.10	0.31	0.04	0.01	0.23	0.46	0.42	0.23
The east mining area	Max	0.52	1.49	127.27	4.19	0.07	14.75	22.32	184.33	12.61	0.92	1.80	0.42
	Min	0.37	0.38	125.85	1.45	0.06	3.88	15.28	181.12	5.38	0.37	0.26	0.22
	Average	0.47	0.94	126.65	2.97	0.06	9.86	17.40	183.02	8.91	0.63	0.95	0.36
	Coefcient of variation	0.11	0.41	0.00	0.35	0.10	0.42	0.15	0.01	0.28	0.35	0.72	0.20

Statistical results are derived from reference 15 and this study.

The Luobei Depression contains the largest single brine deposit of potassium sulphate in the world, and includes three sub-mineral areas: the Xinqing Platform, Luobei Depression, and Tenglong Platform, from west to east (Fig. 1)^22^. The Luobei Depression sub-mineral area is the main storage site for potassium-rich brine, with seven brine reservoirs at a shallow depth of 200 m, including one layer of phreatic brine and six layers of confined water; the grade of KCl varies from 1.2 to 1.45%^23^. The potassium-rich brine deposits are mainly hosted in intergranular glauberite [Na_2_Ca(SO_4_)_2_], and the hydrochemical type is the magnesium sulphate subtype, with an average salinity of 367 g/L, slightly higher than the phreatic brine in the west mining area of Dawadi. The total average salinity is 341.33 g/L in the Xinqing, Tenglong, and Luobei sub-mineral areas^23^, which is comparable to the brine in the east mining area of Dawadi (341.37 g/L).

**Figure 2:**
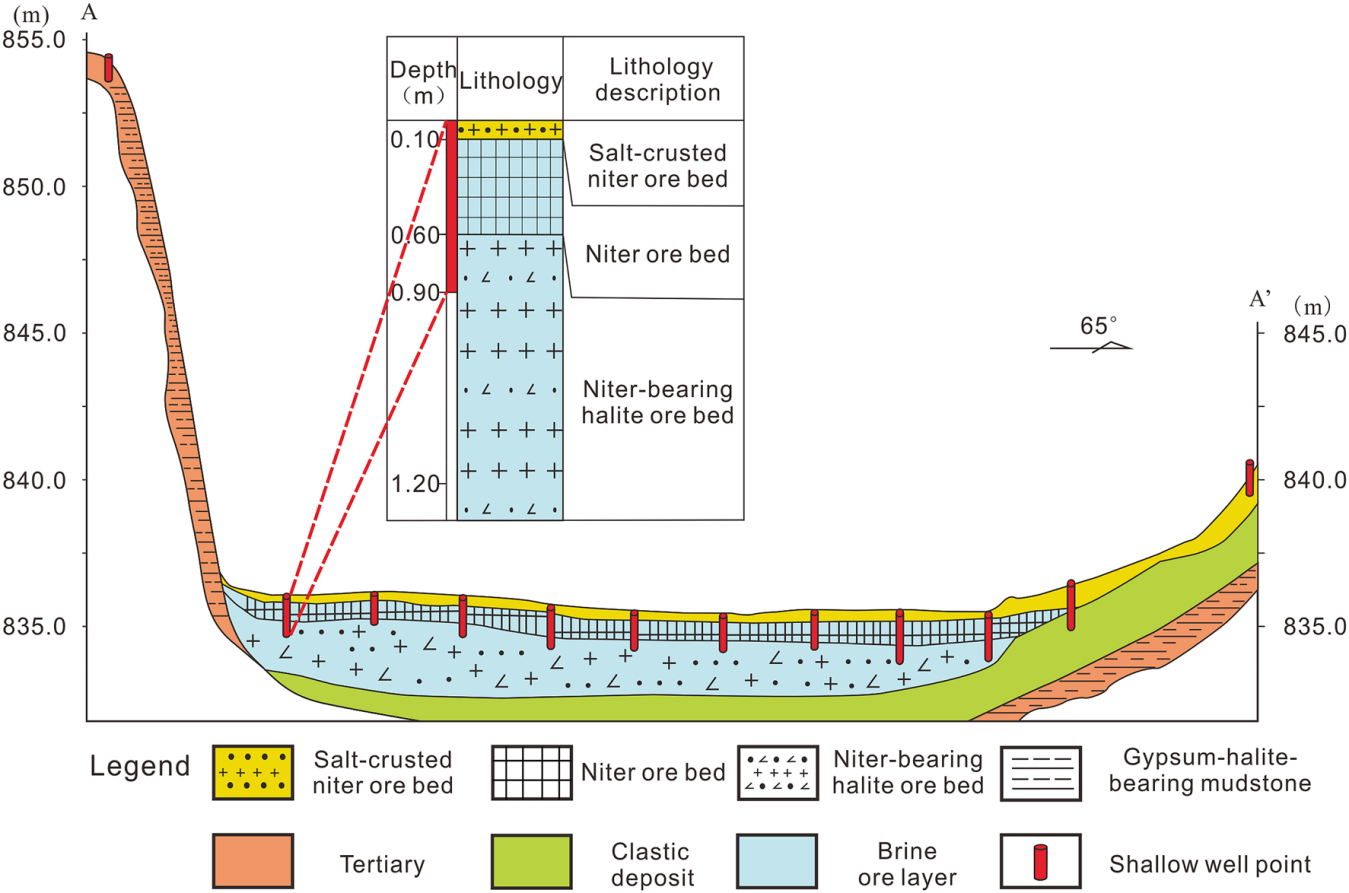
Shallow well section in the west mining area of Dawadi^16^.

In the solid-phase deposit of the west mining area of Dawadi, from top to bottom, there are a salt-crusted potassium nitrate ore layer, a potassium nitrate ore layer, and a potassium nitrate-bearing halite ore layer^15^; the solid-phase potassium nitrate ore layer is mainly found in the upper part of the ore body, with a thickness of about 0.60–0.90 m, accounting for only 11%–15% of the thickness of the salt deposits. The vertical and horizontal profiles of the ore body are shown in Fig. 2, and the location is shown in Fig. 1. From the top to the bottom of the ore body, the mineral size gradually increased, the density gradually decreased, and the water content gradually increased. The salt minerals mainly included nitre (KNO_3_), nitronatrite (NaNO_3_), halite (NaCl), humberstonite [K_3_Na_7_Mg_2_(SO_4_)_6_(NO_3_)_2_·6H_2_O], gypsum (CaSO_4_·2H_2_O), thenardite (Na_2_SO_4_), and salt cake (Na_2_SO_4_·10H_2_O). The sampling points of the solid-phase ore layer are shown in Fig. 1, and the chemical compositions are shown in Table 2; some ores contained much higher KNO_3_ contents than the industrial grade of 3.5%^1^.

**Table 2:** Chemical compositions of the solid potassium nitrate deposit in Dawadi.

		H_2_O^−^ (%)	Insoluble matter (%)	CaSO_4_ (%)	MgSO_4_ (%)	Na_2_SO_4_ (%)	NaCl (%)	NaNO_3_ (%)	KNO_3_ (%)	KCl (%)
The west mining area	Max	0.66	64.85	5.30	3.51	24.64	78.97	12.23	14.37	1.46
	Min	0.17	4.56	0.61	0.10	2.95	21.40	0.00	0.72	0.00
	Average	0.39	31.12	2.09	1.69	8.30	48.55	4.35	6.66	0.20
	Coefcient of variation	0.40	0.54	0.62	0.72	0.75	0.41	0.91	0.57	2.42
The east mining area	Max	0.56	40.95	4.08	1.14	3.11	87.72	0.22	1.88	0.50
	Min	0.11	8.51	0.07	0.13	0.00	52.83	0.00	0.09	0.00
	Average	0.30	18.85	1.81	0.62	1.18	75.42	0.03	0.51	0.09
	Coefcient of variation	0.45	0.48	0.65	0.53	0.79	0.14	2.12	1.00	1.31

Statistical results are derived from reference 15 and this study.

### Source of ore-forming materials of Dawadi potassium nitrate deposit

Previous studies on the sources of mineral-forming substances in nitrate deposits focused on the sources of nitrogen and the formation mechanism of NO_3_^−^^5–7^. Natural nitrogen is mainly attributed to the atmosphere (4 × 10^18^ kg·N), oceans (2.4 × 10^16^ kg·N), biomass (5 × 10^11^ kg·N), and bulk silicate Earth (27 ± 16 × 10^18^ kg·N)^24^. Yoshioka et al.^25^ considered the nitrogen storage capacity of the mantle to be at least 53 times higher than that of the atmosphere, based on the solubility of nitrogen. Moreover, there are two main ways nitrate is formed: microbial nitrification and atmospheric photochemical reactions. Because nitrate from different sources and different mechanisms has different nitrogen and oxygen isotopic signatures^26^, nitrogen and oxygen isotopes have been important means to trace the sources and transport mechanisms of mineralized material.

**Figure 3:**
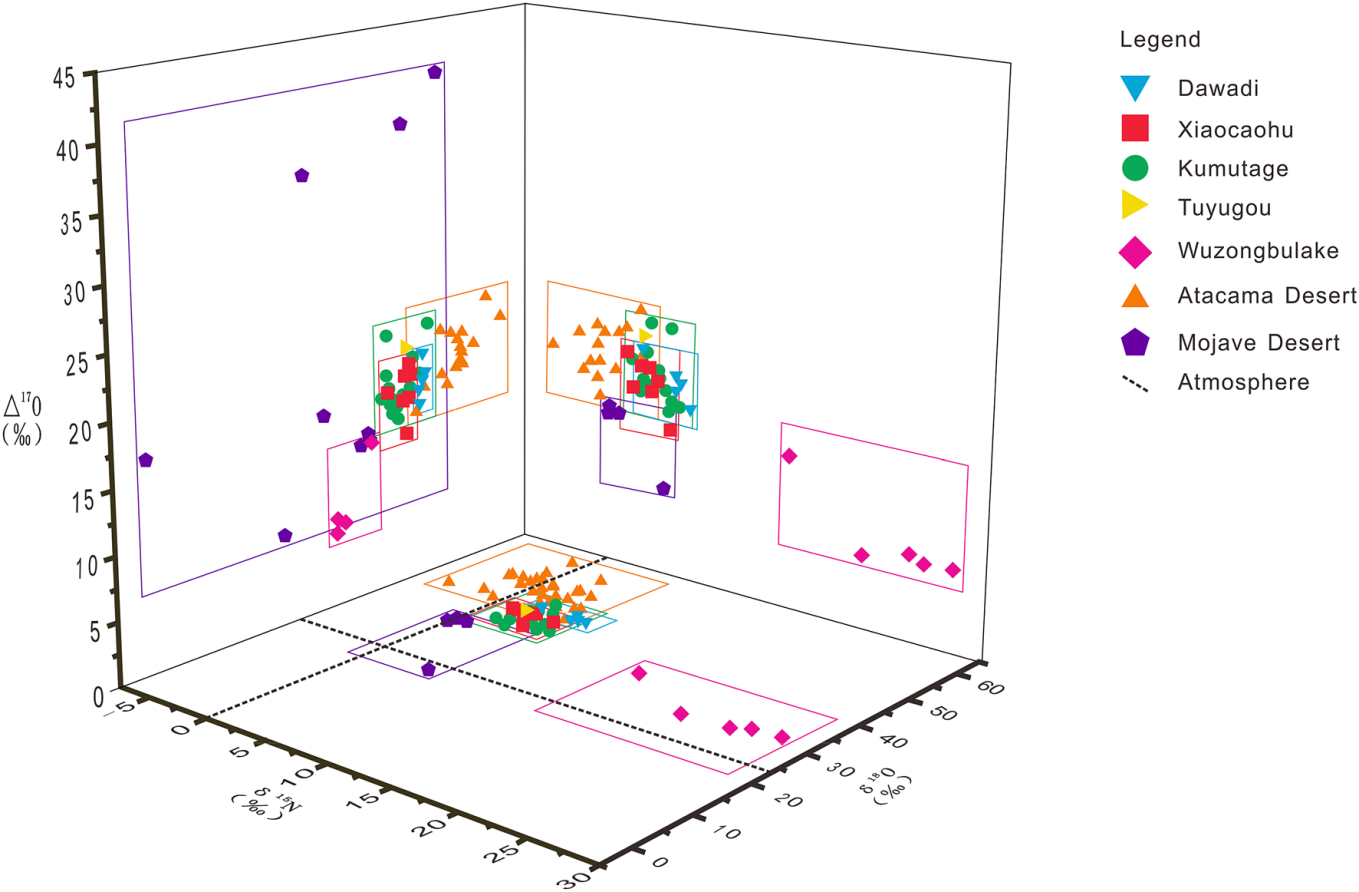
The casting point of oxygen and nitrogen stable isotope signatures of Dawadi, in comparison with those from the Turpan-Hami area, Mojave Desert, and Atacama Desert. (N and O isotope values of nitrate deposits in the Mojave Desert and Atacama Desert according to references 5, 7, 9, 10, and 12; N and O isotopes of nitrate deposits in Dawadi, Xiaocaohu, Kumutage, Tuyugou, and Wuzongbulake, according to reference 7).

The results of nitrogen and oxygen isotope tests of the Dawadi samples show that the δ^15^N_Air_[NO_3_^−^] values ranged from 2.9‰ to 6.9‰^7^, with a mean value of 5.46‰, which is slightly higher than those of the Atacama nitrate deposit and Mojave nitrate deposit^5^, with values of − 4.8‰ to 4.2‰ and 0.2‰ to 4.9‰ respectively. These values are also higher than those of sodium-nitrate deposits in the Turpan-Hami Basin, but lower than that of Wuzongbulake (Fig. 3). They are also located in the middle and upper part of the δ^15^N values of − 5‰ to 5‰^27^ for atmospheric photochemical genesis of nitrate, as shown in Fig. 3. The δ^18^O_V-SMOW_[NO_3_^−^] values of Dawadi were from 44.9 to 46.0‰^7^, with a mean value of 45.38‰, higher than that of atmospheric δ^18^O_V-SMOW_ (+ 23.5‰), below the average of rainwater in the outer suburbs of Colorado (δ^18^O_V-SMOW_[NO_3_^−^] = 40‰ to 70‰)^14^, and below the values of atmospheric deposition (δ^18^O_V-SMOW_[NO_3_^−^] = 53‰ to 73‰)^13^. The δ^18^O_V-SMOW_[NO_3_^−^] values of 35.6‰ to 50.4‰ partially overlap with those of the Atacama Desert nitrate deposits, but are higher than those of the Mojave nitrate deposits (δ^18^O_V-SMOW_[NO_3_^−^] = 21.3‰ to 33.1‰)^5^, similar to those of the sodium-nitrate deposits in the Turpan-Hami area, and significantly higher than those of the potassium nitrate deposits in Wuzongbulake. In addition, the Δ^17^O[NO_3_^−^] values of Dawadi were from 14.3‰ to 18.4‰^7^, with a mean value of 16.24‰, indicating obvious oxygen isotope non-mass fractionation, which suggests that the origin of the deposit was related to atmospheric photochemical reactions. At the same time, previous research suggested that the sodium-nitrate deposits in the northern Turpan-Hami area, in contrast, were mainly formed by long-term deposition of atmospheric nitrate aerosols^7,11^.

However, atmospheric deposition cannot explain the development of typical sulphate-type potash deposits in the Luobei Depression in Lop Nor, which is located south of the Turpan-Hami Basin and has similar climatic conditions. Moreover, the Luobei Depression contains almost no nitrate, with an average of only 9.75 mg/L^19^, which is even lower than the NO_3_^−^ content of 30 mg/L in marine water^28^; yet the salinity of brine in the Luobei Depression is as high as 367 g/L^23^, 10 times higher than that of seawater. Therefore, large-scale systematic sampling and analysis of salt springs and groundwater in the Lop Nor and Dawadi areas were conducted to explore the hydrological connections between the Dawadi Depression and other sub-depressions of the Lop Nor region and the relationship between the Luobei Depression and the genesis of the Dawadi potassium nitrate deposit.

From north to south, the topography of the Dawadi and Luobei depressions and Great Ear Lake decrease in turn, separated by the uplift, forming three secondary depressions (Fig. 1). The results of chemical analysis show that the concentrations of nitrate ions in the brine of the Dawadi nitrate mine area were from 3,880 to 36,210 mg/L, with an average value of 16,505 mg/L. The nitrate content of subsurface phreatic water (clastic reservoir) at the perimeter of the mine area varied from 495 to 16,719 mg/L, with an average value of 4,706.43 mg/L. The nitrate contents of salt springs at the edge of the basin were from 223 to 8,320 mg/L, with an average value of 3,080.82 mg/L. In contrast, the nitrate content in the Luobei Depression was 1–35 mg/L, with an average of only 9.75 mg/L^19^; the nitrate content in the Great Ear Lake area in the southern part of Lop Nor ranged from 2.70 to 102 mg/L, with an average value of 37.28 mg/L; and the nitrate contents in the Aqike Depression of eastern Lop Nor ranged from 0.87 to 2.76 mg/L, with an average value of only 1.82 mg/L (Table 3). These results show that compared with those of other depressions in Lop Nor, the groundwater and surrounding exposed salt springs in the Dawadi Depression are super-enriched in NO_3_^−^, which also indicates that the groundwater systems in the Dawadi Depression and the adjacent Luobei Depression are independent and not connected to each other. Zhang et al.^1^ also reported high NO_3_^−^ contents in the Dawadi area, with NO_3_^−^ of 159 mg/L detected in the Jianquanzi area to the north of the Dawadi Depression and NO_3_^−^ of up to 3,600 mg/L in the Aqishan brine, which is significantly higher than the NO_3_^−^ contents of 7 mg/L in surface river water in arid regions of West China, 8–30 mg/L in shallow groundwater, and 4–35 mg/L in spring water^29^, but much lower than the ultra-high anomalous salt spring sites found in this study, with NO_3_^−^ contents of 8,000 mg/L or greater.

**Table 3:** Nitrate concentration of brine in Lop Nor (ρ(B)/(mg/L)).

	Max	Min	Average	Coefcient of variation
Dawadi mining area	36,210	3880	16,505	0.37
Underground phreatic water around the Dawadi mining area	16,719	495	4706.43	1.28
Basin margin salt spring	8320	223	3080.82	1.02
Luobei depression	35	1	9.75	1.14
Great Ear area	102	2.70	37.28	0.96
Aqike depression	2.76	0.87	1.82	0.74

Statistical results for the Dawadi mine area were derived from reference 15 and the results of this study; those for the Luobei Depression were derived from reference 19; data for underground phreatic water around the Dawadi mining area, basin margin salt spring, the Great Ear area, and the Aqike Depression are the results of this study.

At the same time, two deep hydrothermal-type (Ca–Cl) brines were found in the salt spring investigation; both were exposed along the fault zone of the basin margin (Fig. 1). Ca–Cl type water is a typical deep-source fluid, with chemical properties that are completely different from those of surface water bodies. The calcium equivalent concentration of the brine was much larger than the sum of carbonate and sulphate and also had considerably higher metal contents (Li, Sr), whereas in the Ca–Cl type brine found in this study, the nitrate content was as high as 8,044 mg/L, indicating that the deep source may have been another important source of nitrate mineralization in the Dawadi deposit. Water–rock interaction with surrounding rocks, such as volcanic rocks, during the uplift of deep hydrothermal fluids along the fracture may have been an important process for nitrogen enrichment. Ge et al.^4^ detected NO_3_^−^ content of 410 mg/L in the rising spring in the Kuzi Mountains in the Turpan-Hami Basin (Fig. 1), and although the water chemistry type was not reported, they considered the deep source to be an important material source of NO_3_^−^ based on field geological investigation.

In the Carboniferous volcanic rocks and Jurassic coal-bearing and oil-bearing strata in the Turpan-Hami Basin, China, the nitrate content is high and has some spatial connection with nitrate deposits; therefore, the organic matter in volcanic rocks and soils formed by volcanic activity around the deposits is considered to be the main source of nitrogen^30,31^. Water–rock reactions and leaching transported nitrogen and other elements from the rock and soil, and these elements ultimately converged to form the deposit. In addition, the distribution of nitrate deposits in the Turpan-Hami Basin is closely related to faults (Fig. 1), which would provide transport channels for NO_3_^−^-rich fluids formed by deep water–rock interactions, creating conditions for fluid migration, convergence, and movement to favorable tectonic locations for mineralization emplacement.

Therefore, the groundwater played an important role in the process of nitrate mineralization in the Dawadi deposit, not only as a transport medium, but also as an important source of the mineralized material.

### Metallogenic model

#### Tectonics

The Tarim Basin was inverted by the end of Neogene because of the Himalayan movement and formed a geomorphological pattern that tilted from southwest to northeast, making Lop Nor the lowest depression of the Tarim Basin^23^. At the same time, the Lop Nor Basin was also subjected to strong north–south extrusion, which cleaved it into different secondary tectonic depressions under the control of fracture tectonics, forming different depressions such as the Luobei Depression and the Dawadi Depression in the north. This provided a closed depositional environment for the formation and preservation of the nitrate deposit.

#### Climate

The Lop Nor region experienced a series of climatic changes between dry and wet conditions during the Quaternary Period, but with the uplift of the Tibetan Plateau, the general trend was toward aridity, with an extremely arid climatic at the end of the Holocene. The average annual temperature in the region is 11.6 °C, with a maximum summer temperature of 50°C^23^, precipitation < 20 mm/y, and evaporation of about 3,500 mm/y^32^, which is a typical continental arid climate; north-easterly winds prevail throughout the year, with an average annual wind speed > 5 m/s^33^, which provided the necessary arid climatic conditions for the formation of the salt-lake type nitrate deposit in Dawadi.

#### Origin and mineralization process

Dawadi is located in the southern part of the potassium-rich nitrate minerogenetic belt of the eastern Tianshan Mountains, which is south of the sodium-nitrate minerogenetic province in the Turpan-Hami Basin and north of the potash minerogenetic province in the Lop Nor Basin (Fig. 1). The nitrate contents in phreatic water and salt springs in Dawadi indicate that groundwater played an important role in the mineralization process in Dawadi, but because Dawadi is in a different geochemical and hydrological system from the Luobei Depression, its source may be closely related to the groundwater system of the potassium-rich nitrate and sodium-nitrate minerogenetic belt to the north.

Because of the extensive distribution of volcanic rocks rich in nitrate in the northern Turpan-Hami Basin^30,31^, the NO_3_^−^-rich fluids formed by groundwater–rock interactions in this region may have been an important source of the nitrate deposits, and transportation, convergence, and evaporation acted to enrich mineralization in the Dawadi Depression in the lowest terrain on the southern edge of the minerogenetic belt, under tectonic as well as topographic driving forces. In addition, this deposit represents the first time that deep hydrothermal (Ca–Cl) brines have been found to be extraordinarily enriched in NO_3_^−^ in a basin margin fracture zone; thus, it provides evidence that the NO_3_^−^ may have been partially derived from the water–rock reactions of deep hydrothermal fluids in the Dawadi Depression. Moreover, the distribution of nitrate deposits in the Turpan-Hami Basin is related to macroscopic fracture spreading (Fig. 1); thus, the deep-sourced brines may play an important role in the nitrate minerogenetic belt of the entire Turpan-Hami Basin. However, there is a lack of systematic investigations and studies on the regional scale of the salt springs and water chemistry types in the fracture zone. Moreover, in addition to conventional atmospheric dry and wet deposition, the early formation of sodium nitrate in the uplands around the Dawadi Depression is also a direct material source^11^, and a portion of the NaNO_3_-rich fluid formed by selective dissolution and leaching by seasonal precipitation enters the lake directly, whereas the other portion is submerged into the subsurface. Under the combined effects of surface water and groundwater from multiple sources, the mineralized material was gradually transported to the Dawadi Depression, which is a closed depression in the southern part of Kuruqtag, forming a nitrate salt lake, and evaporating under the influence of the arid climate to form solid-phase minerals such as nitre, nitronatrite, halite, humberstonite, and salt cake, ultimately forming evaporative depositional solid–liquid coexistence in the salt-lake type potassium nitrate deposit.

**Figure 4:**
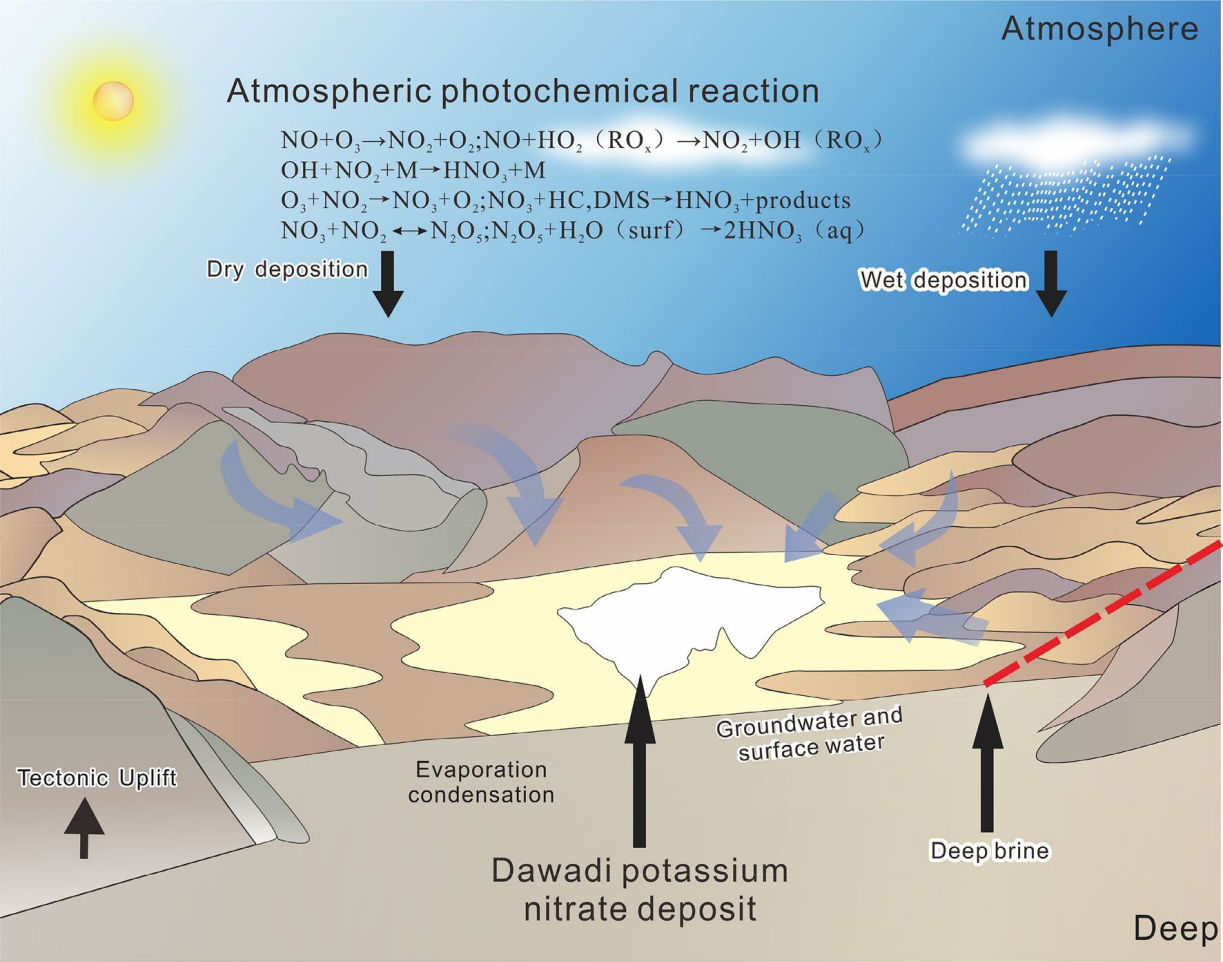
Schematic diagram of the proposed metallogenic model of the Dawadi potassium nitrate deposit^34^.

This study provides a new perspective on the material source and mineralization process of the Dawadi potassium nitrate deposit and proposes a multi-source mineralization model for this deposit (Fig. 4).

## Conclusions

There is a large sulphate-type potassium-rich brine deposit in the Luobei Depression, a sub-depression of the Lop Nor Basin, whereas there is a rare potassium nitrate deposit in the adjacent Dawadi Depression. In this report, a detailed comparison of the characteristics of the deposits in these two sub-depressions and a basin-wide hydrochemical analysis are presented to explore the hydrological link between the Dawadi and Luobei depressions and the source of the potassium nitrate deposit in the Dawadi Depression.

1. The Dawadi potassium nitrate deposit is divided into two mining area, east and west. In the east mining area, the KNO_3_ content in the solid-phase ore body is 0.09%–1.88%, which does not reach industrial grade, and the NO_3_^−^ in the confined brine reaches 3,880–14,750 mg/L. In the west mining area, the KNO_3_ content in the solid-phase ore body is 0.72%–14.37%, and the NO_3_^−^ in the brine ore body reaches 13,570–36,210 mg/L, both of which are higher than the threshold for industrial grade. The average salinity of the brine in the Dawadi Depression is 352.07 g/L, and this brine is hosted intergranularly in halite and salt cake layers. In contrast, the Luobei Depression is a sulphate-type potash deposit, with the exception of a thin layer of solid polyhalite deposits at the top of the formation, and the main ore deposit is potassium-rich brine, with an average salinity of 367 g/L and a KCl grade varying from 1.2% to 1.45%, mainly in the intergranular glauberite.
2. The investigation showed that the nitrate was highly enriched in the phreatic water around the Dawadi mining area and the salt springs at the basin edge, with average contents of 4,706.43 mg/L and 3,080.82 mg/L, respectively, whereas the nitrate contents in the Luobei Depression, the Great Ear area, and the Aqike Depression in the south were 9.75 mg/L, 37.28 mg/L, and 1.82 mg/L, respectively, indicating that the Dawadi and Luobei depressions belong to different groundwater systems that are not connected to each other.
3. A multi-source mineralization model of the Dawadi potassium nitrate deposit was established, suggesting that groundwater played an important role in the nitrate mineralization process in Dawadi, and that groundwater reacted with source rocks such as volcanic rocks to form mineral-rich fluids, which rose along faults and fracture zones and evaporated at the surface to enrich mineralization. In addition, deep hydrothermal (Ca–Cl) salt springs are exposed along fractures at the basin margin, and are super-enriched in nitrate, suggesting that the Dawadi potassium nitrate deposit may have been recharged by deep hydrothermal fluids that reacted with the surrounding rocks, migrated, and rose along the fractures to cause enrichment and mineralization. In addition to conventional atmospheric dry and wet deposition, the early formation of sodium nitrate in the surrounding uplands of the Dawadi Depression may also have provided a direct source. Under the combined effect of multiple sources, a rare solid–liquid salt-lake type potassium nitrate deposit was formed in the Dawadi Depression, Lop Nor.
